# Polydeoxyribonucleotide Ameliorates Lipopolysaccharide-Induced Lung Injury by Inhibiting Apoptotic Cell Death in Rats

**DOI:** 10.3390/ijms18091847

**Published:** 2017-08-24

**Authors:** Jin An, So Hee Park, Il-Gyu Ko, Jun-Jang Jin, Lakkyong Hwang, Eun-Sang Ji, Sang-Hoon Kim, Chang-Ju Kim, So Young Park, Jae-Joon Hwang, Cheon Woong Choi

**Affiliations:** 1 Department of Pulmonary and Critical Care Medicine, Kyung Hee University Hospital at Gangdong, Seoul 05278, Korea; anjin7487@gmail.com (J.A.); sojjang01@gmail.com (S.H.P.); 2 Department of Physiology, College of Medicine, Kyung Hee University, Seoul 02447, Korea; rhdlfrb@naver.com (I.-G.K.); threej09@hanmail.net (J.-J.J.); LHWANGPHD@gmail.com (L.H.); wldmstkd11@hanmail.net (E.S.J.); spdlvcjstkd@naver.com (S.-H.K.); changju@khu.ac.kr (C.-J.K.); 3 Department of Pulmonary and Critical Care Medicine, Kyung Hee University Medical Center, Seoul 05278, Korea; sy.park12@gmail.com; 4 Department of Internal Medicine, College of Medicine, Kyung Hee University, Seoul 05278, Korea; hjjoon00@naver.com

**Keywords:** lung injury, lipopolysaccharide, polydexyribonucleotide, apoptosis, adenosine A_2A_ receptor

## Abstract

Lung injury is characterized by diffuse lung inflammation, alveolar-capillary destruction, and alveolar flooding, resulting in respiratory failure. Polydexyribonucleotide (PDRN) has an anti-inflammatory effect, decreasing inflammatory cytokines, and suppressing apoptosis. Thus, we investigated its efficacy in the treatment of lung injury, which was induced in rats using lipopolysaccharide (LPS). Rats were randomly divided into three groups according to sacrifice time, and each group split into control, lung injury-induced, and lung injury-induced + PDRN-treated groups. Rats were sacrificed 24 h and 72 h after PDRN administration, according to each group. Lung injury was induced by intratracheal instillation of LPS (5 mg/kg) in 0.2 mL saline. Rats in PDRN-treated groups received a single intraperitoneal injection of 0.3 mL distilled water including PDRN (8 mg/kg), 1 h after lung injury induction. Percentages of terminal deoxynucleotidyl transferase-mediated dUTP nick end labeling (TUNEL)-positive, cleaved caspase-3-, -8-, and -9-positive cells, the ratio of Bcl-2-associated X protein (Bax) to B-cell lymphoma 2 (Bcl-2), and expressions of inflammatory cytokines (tumor necrosis factor-α, interleukin-6) were decreased by PDRN treatment in the LPS-induced lung injury rats. Therefore, treatment with PDRN reduced lung injury score. This anti-apoptotic effect of PDRN can be ascribed to the enhancing effect of PDRN on adenosine A_2A_ receptor expression. Based on these results, PDRN might be considered as a new therapeutic agent for the treatment of lung injury.

## 1. Introduction

Acute lung injury (ALI) is characterized by disruption of the alveolar-capillary membrane barrier and resultant pulmonary edema, and is associated with proteinaceous alveolar exudate [1]. Mortality from ALI decreased in the past decade, due in part to the implementation of lung-protective ventilation strategies, however, ALI-related lethality remains high [2]. Thus, new pharmacological therapies based on the ALI pathogenesis are needed. 

The pathophysiological mechanisms of ALI are complex and this disease is caused by various factors. Among them, endotoxins are the most important pathogenic component [3,4]. Cytokine-mediated inflammation is implicated in the pathogenesis of ALI [5]. Increased local and systemic inflammatory mediators such as tumor necrosis factor-α (TNF-α), interleukin-6 (IL-6), and activated leukocytes may cause systemic inflammation [6]. Furthermore, increasing evidence suggests that apoptosis also plays an important role in the progression of ALI [4,7].

Apoptosis represents one form of cell death including autophagic cell death and autonomous necrosis. Apoptosis is a mechanism to remove excessively damaged or potentially harmful cells to maintain normal cellular homeostasis [8,9]. Although apoptosis is a ‘clean’ form of cell death, apoptotic cells that are not rapidly removed eventually undergo secondary necrosis associated with leakage of cellular content and inflammation, leading to severe tissue damage [10]. Tang et al. [11] reported that alveolar cell apoptosis likely contributes to ALI in response to various environmental stimuli by inducing endothelial and epithelial barrier dysfunction. DNA fragmentation that is characteristic of apoptotic cell death is detected by terminal deoxynucleotidyl transferase-mediated dUTP nick end labeling (TUNEL) assay [12]. Caspases are a family of proteases that play an essential role in programmed cell death and inflammation. Caspase-3 is a key executor of apoptosis, whereas caspase-8 and caspase-9 are the initiator caspases and they are most likely to act on caspase-3 [13]. The cleaved forms of caspases activate pro-apoptotic pathways leading to DNA degradation and cell death [13]. In addition to caspases, B-cell lymphoma 2 (Bcl-2) family proteins also play an important role in the regulation of apoptosis. Bcl-2 family proteins are classified as anti-apoptotic proteins including Bcl-2 and Bcl-_XL_, and pro-apoptotic proteins, such as Bcl-2-associated X protein (Bax) and BH3 interacting-domain death agonist (Bid). The balance between pro-apoptotic and anti-apoptotic Bcl-2 family members determines the mitochondrial response to apoptotic stimuli [14]. The imbalance between pro-apoptotic and anti-apoptotic mediators causes apoptosis and increases susceptibility to lung injury [15].

The actions of adenosine are mediated through the following G protein-coupled receptors, namely A_1_, A_2A_, A_2B_, and A_3_, which are expressed diversely in immune cells [16]. Among them, the adenosine A_2A_ receptor is found in most cells associated with wound healing [17]. Polydeoxyribonucleotide (PDRN), extracted from the sperm of salmon, has been shown to stimulate tissue repair in chronic wounds and burns [18]. PDRN stimulates vascular endothelial growth factor expression by activating the adenosine A_2A_ receptor [19,20]. PDRN has also been shown to inhibit apoptosis and inflammation in the experimental gastric ulcer [20]. 

Although PDRN is known to promote wound healing and suppress apoptotic cell death, the effects of PDRN on lung injury have not been reported. In the present study, we investigated the effect of PDRN treatment on lipopolysaccharide (LPS)-induced lung injury using rats. For this study, analysis of lung injury score was performed by hematoxylin and eosin (H&E) staining. Additionally, TUNEL assay, immunohistochemistry for cleaved caspase-3, -8, -9, and Western blotting for Bax, Bcl-2, TNF-α, IL-6, and adenosine A_2A_ receptors were performed.

## 2. Results 

### 2.1. Effect of Polydexyribonucleotide (PDRN) on Histological Alteration and Lung Injury Score

**Figure 1:**
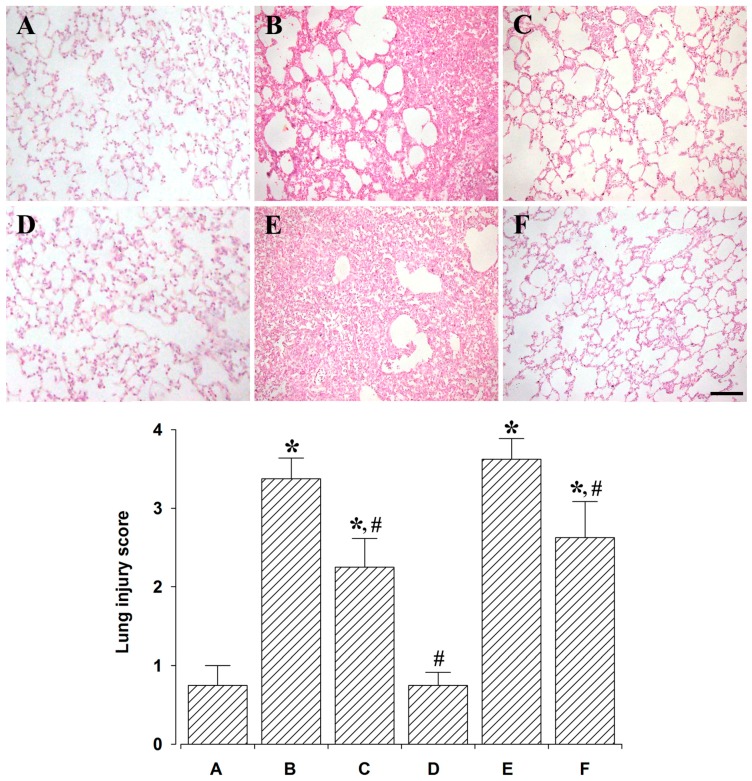
Effect of polydeoxyribonucleotide (PDRN) treatment on the lung injury score. **Upper**: Photomicrographs of lung injury. The scale bar represents 100 μm. (**A**) Control and 24 h after sacrifice group; (**B**) Lung injury and 24 h after sacrifice group; (**C**) Lung injury with PDRN-treatment and 24 h after sacrifice group; (**D**) Control and 72 h after sacrifice group; (**E**) Lung injury and 72 h after sacrifice group; (**F**) Lung injury with PDRN-treatment and 72 h after sacrifice group. **Lower**: Lung injury score in each group. * represents *p* < 0.05 compared to the control and 24 h after sacrifice group. ^#^ represents *p* < 0.05 compared to the lung injury and 24 h after sacrifice group.

Histological alterations and lung injury scores are presented in Figure 1. At 24 h after LPS administration, intra-alveolar hemorrhage and fibrin, interstitial edema, and acute and chronic inflammatory cell infiltration moderately occupying the alveolar lumen were seen. At 72 h after LPS administration, intra-alveolar hemorrhage and fibrin, interstitial edema, and acute and chronic inflammatory cell infiltration severely occupying the alveolar lumen were seen. However, patch intra-alveolar macrophages were seen 24 h after PDRN treatment and normal-looking alveolar structures, except type II pneumocytes hyperplasia were observed 72 h after PDRN treatment.

The lung injury scores were 0.75 ± 0.25 in the control and 24 h after sacrifice group, 3.37 ± 0.26 in the lung injury and 24 h after sacrifice group, 2.25 ± 0.36 in the lung injury with PDRN-treatment and 24 h after sacrifice group, 0.78 ± 0.22 in the control and 72 h after sacrifice group, 3.62 ± 0.26 in the lung injury and 72 h after sacrifice group, and 2.62 ± 0.46 in the lung injury with PDRN-treatment and 72 h after sacrifice group.

These results indicate that lung injury score was significantly increased by the induction of lung injury (*p* < 0.05), whereas, PDRN treatment significantly decreased lung injury score (*p* < 0.05). 

### 2.2. Effect of PDRN on Percentage of Terminal Deoxynucleotidyl Transferase-Mediated dUTP Nick End Labeling (TUNEL)-Positive Cells 

**Figure 2:**
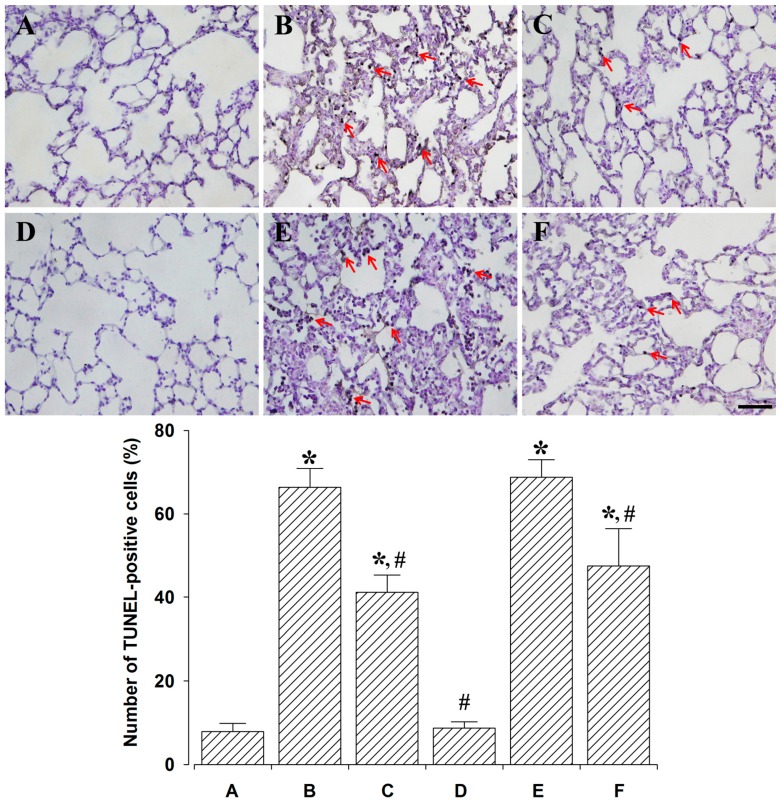
Effect of polydeoxyribonucleotide (PDRN) treatment on the percentage of terminal deoxynucleotidyl transferase-mediated dUTP nick end labeling (TUNEL)-positive cells. **Upper**: Photomicrographs of TUNEL-positive cells. The scale bar represents 100 μm. Red arrows represent TUNEL-positive cells. (**A**) Control and 24 h after sacrifice group; (**B**) Lung injury and 24 h after sacrifice group; (**C**) Lung injury with PDRN-treatment and 24 h after sacrifice group; (**D**) Control and 72 h after sacrifice group; (**E**) Lung injury and 72 h after sacrifice group; (**F**) Lung injury with PDRN-treatment and 72 h after sacrifice group. **Lower**: Percentages of TUNEL-positive cells. * represents *p* < 0.05 compared to the control and 24 h after sacrifice group. ^#^ represents *p* < 0.05 compared to the lung injury and 24 h after sacrifice group.

Photomicrographs of TUNEL-positive cells in the lung tissues are shown in Figure 2. Percentages of TUNEL-positive cells were 7.87 ± 1.96% in the control and 24 h after sacrifice group, 66.37 ± 4.51% in the lung injury and 24 h after sacrifice group, 41.12 ± 4.13% in the lung injury with PDRN-treatment and 24 h after sacrifice group, 8.75 ± 1.47% in the control and 72 h after sacrifice group, 68.75 ± 4.21% in the lung injury and 72 h after sacrifice group, 47.50 ± 8.93% in the lung injury with PDRN-treatment and 72 h after sacrifice group.

These results indicate that DNA fragmentation was significantly increased by the induction of lung injury (*p* < 0.05), whereas PDRN treatment significantly decreased DNA fragmentation (*p* < 0.05). 

### 2.3. Effect of PDRN on Percentages of Cleaved Caspase-3-, -8-, and -9-Positive Cells 

**Figure 3:**
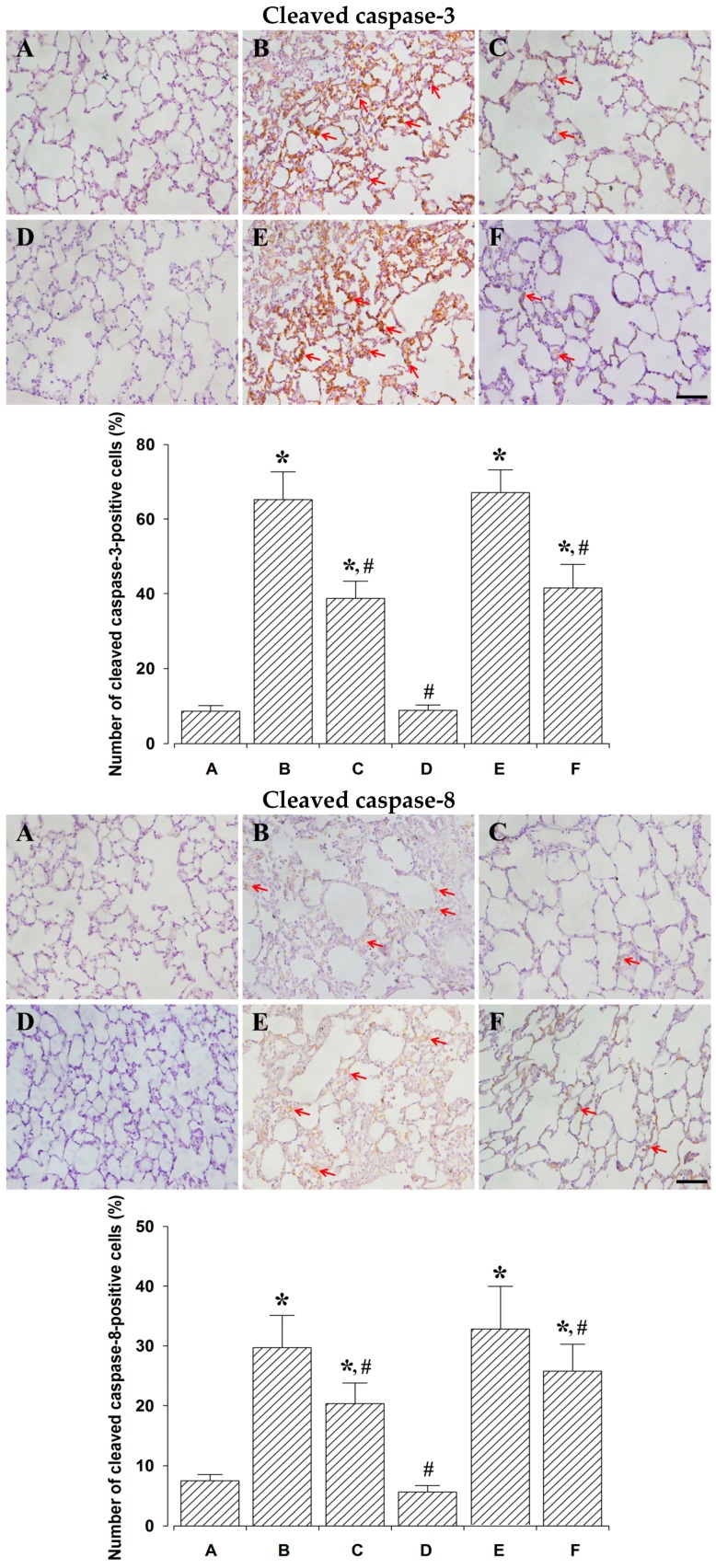

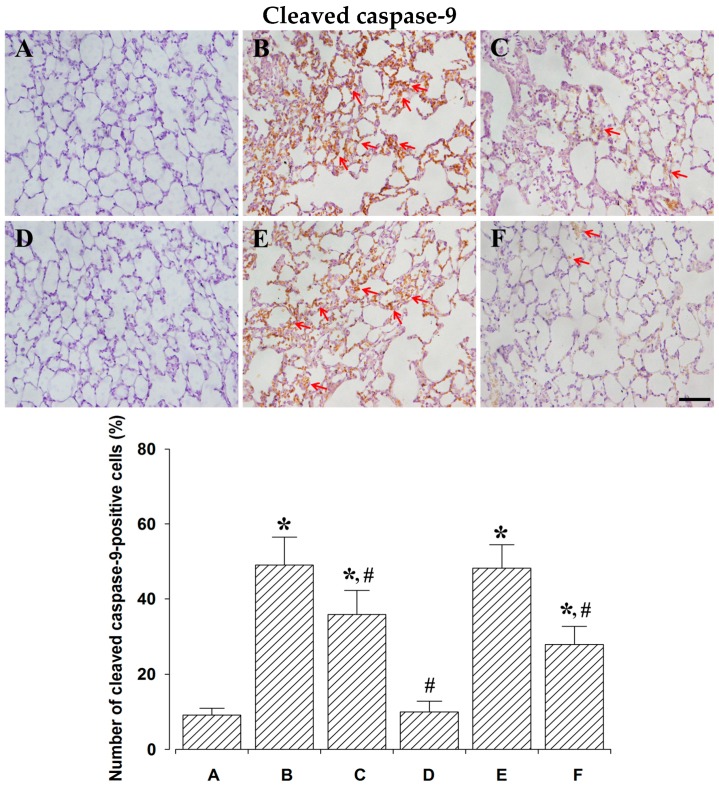
The effect of polydeoxyribonucleotide (PDRN) treatment on the percentages of cleaved caspase-3-, -8-, -9-positive cells. **Upper**: Cleaved caspase-3-positive cells. (**Top**) Photomicrographs of cleaved caspase-3-positive cells. (**Down**) Percentages of cleaved caspase-3-positive cells in each group. Middle: Cleaved caspase-8-positive cells. (**Top**) Photomicrographs of cleaved caspase-8-positive cells. (**Down**) Percentages of cleaved caspase-8-positive cells in each group. Lower: Cleaved caspase-9-positive cells. (**Top**) Photomicrographs of cleaved caspase-9-positive cells. (**Down**) Percentages of cleaved caspase-9-positive cells in each group. The scale bar represents 100 μm. Red arrows represent cleaved caspase-positive cells. (**A**) Control and 24 h after sacrifice group; (**B**) lung injury and 24 h after sacrifice group; (**C**) lung injury with PDRN-treatment and 24 h after sacrifice group; (**D**) control and 72 h after sacrifice group; (**E**) lung injury and 72 h after sacrifice group; (**F**) lung injury with PDRN-treatment and 72 h after sacrifice group. * represents *p* < 0.05 compared to the control and 24 h after sacrifice group. ^#^ represents *p* < 0.05 compared to the lung injury and 24 h after sacrifice group.

Photomicrographs of cleaved caspase-3-, -8-, and -9-positive cells are presented in Figure 3. The percentage of caspase-3-positive cells was 8.62 ± 1.52% in the control and 24 h after sacrifice group, 62.25 ± 7.44% in the lung injury and 24 h after sacrifice group, 38.75 ± 4.57% in the lung injury with PDRN-treatment and 24 h after sacrifice group, 8.87 ± 1.39% in the control and 72 h after sacrifice group, 67.12 ± 6.11% in the lung injury and 72 h after sacrifice group, 41.50 ± 6.37% in the lung injury with PDRN-treatment and 72 h after sacrifice group. 

Percentage of cleaved caspase-8-positive cells was 7.50 ± 1.05% in the control and 24 h after sacrifice group, 29.87 ± 5.40% in the lung injury and 24 h after sacrifice group, 20.37 ± 3.40% in the lung injury with PDRN-treatment and 24 h after sacrifice group, 5.62 ± 1.06% in the control and 72 h after sacrifice group, 33.12 ± 7.15% in the lung injury and 72 h after sacrifice group, 25.75 ± 4.53% in the lung injury with PDRN-treatment and 72 h after sacrifice group. 

Percentage of cleaved caspase-9-positive cells was 9.12 ± 1.80% in the control and 24 h after sacrifice group, 49.00 ± 7.52% in the lung injury and 24 h after sacrifice group, 35.87 ± 6.37% in the lung injury with PDRN-treatment and 24 h after sacrifice group, 10.00 ± 2.82% in the control and 72 h after sacrifice group, 48.25 ± 6.19% in the lung injury and 72 h after sacrifice group, 27.87 ± 4.80% in the lung injury with PDRN-treatment and 72 h after sacrifice group. 

These results indicate that cleaved caspase-3, -8, and -9 expressions were significantly increased by the induction of lung injury (*p* < 0.05), whereas, PDRN treatment significantly decreased cleaved caspase-3, -8, and -9 expressions (*p* < 0.05).

### 2.4. Effects of PDRN on Expressions of Bax and Bcl-2 

**Figure 4:**
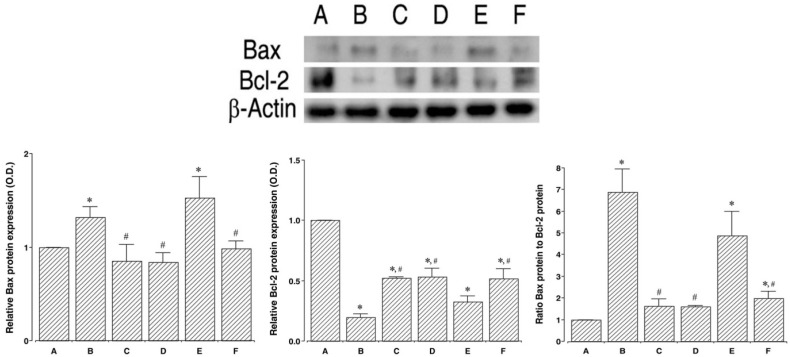
Effect of polydeoxyribonucleotide (PDRN) treatment on the Bcl-2-associated X protein (Bax) and B-cell lymphoma 2 (Bcl-2) expressions. Actin was used as an internal control (46 kDa). **Upper**: The results of band detection using the enhanced chemiluminescence (ECL) detection kit. Groups are labeled as follows: (**A**) Control and 24 h after sacrifice group; (**B**) Lung injury and 24 h after sacrifice group; (**C**) Lung injury with PDRN-treatment and 24 h after sacrifice group; (**D**) Control and 72 h after sacrifice group; (**E**) Lung injury and 72 h after sacrifice group; and (**F**) Lung injury with PDRN-treatment and 72 h after sacrifice group. **Lower**: The relative expressions of Bax and Bcl-2 in each group. * represents *p* < 0.05 compared to the control and 24 h after sacrifice group. ^#^ represents *p* < 0.05 compared to the lung injury and 24 h after sacrifice group.

To verify the effect of PDRN on the expression of apoptotic proteins, the relative expressions of Bax and Bcl-2 were ascertained (Figure 4). When the level of Bax (24 kDa) in the control and 24 h after sacrifice group was set at 1.00, the level of Bax was 1.31 ± 0.11 in the lung injury and 24 h after sacrifice group, 0.85 ± 0.17 in the lung injury with PDRN-treatment and 24 h after sacrifice group, 0.84 ± 0.09 in the control and 72 h after sacrifice group, 1.52 ± 0.22 in the lung injury and 72 h after sacrifice group, 0.98 ± 0.08 in the lung injury with PDRN-treatment and 72 h after sacrifice group. 

When the level of Bcl-2 (26–29 kDa) in the control and 24 h after sacrifice group was set at 1.00, the level of Bax was 0.19 ± 0.02 in the in the lung injury and 24 h after sacrifice group, 0.52 ± 0.00 in the lung injury with PDRN-treatment and 24 h after sacrifice group, 0.53 ± 0.07 in the control and 72 h after sacrifice group, 0.32 ± 0.04 in the lung injury and 72 h after sacrifice group, 0.51 ± 0.08 in the lung injury with PDRN-treatment and 72 h after sacrifice group.

When the ratio of Bax to Bcl-2 in the control and 24 h after sacrifice group was set at 1.00, the ratio of Bax to Bcl-2 was 6.88 ± 1.07 in the in the lung injury and 24 h after sacrifice group, 1.61 ± 0.32 in the lung injury with PDRN-treatment and 24 h after sacrifice group, 1.59 ± 0.05 in the control and 72 h after sacrifice group, 4.88 ± 1.11 in the lung injury and 72 h after sacrifice group, 1.98 ± 0.31 in the lung injury with PDRN-treatment and 72 h after sacrifice group.

These results indicate that induction of lung injury enhanced Bax expression and inhibited Bcl-2 expression (*p* < 0.05), resulting in enhanced Bax to Bcl-2 ratio (*p* < 0.05). However, PDRN treatment suppressed Bax expression and enhanced Bcl-2 expression of (*p* < 0.05), resulting in suppressed Bax to Bcl-2 ratio (*p* < 0.05).

### 2.5. Effect of PDRN on Adenosine A_2A_ Receptor and Inflammatory Cytokines Expressions 

**Figure 5:**
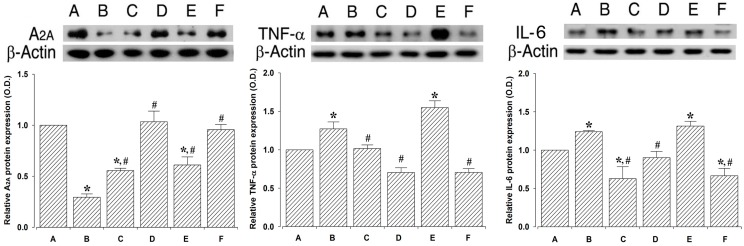
Effect of polydeoxyribonucleotide (PDRN) treatment on the adenosine A_2A_ receptor, tumor necrosis factor-α (TNF-α), and interleukin-6 (IL-6) expressions. Actin was used as an internal control (46 kDa). **Upper**: The results of ban detection using the enhanced chemiluminescence (ECL) detection kit. (**A**) Control and 24 h after sacrifice group; (**B**) Lung injury and 24 h after sacrifice group; (**C**) Lung injury with PDRN-treatment and 24 h after sacrifice group; (**D**) Control and 72 h after sacrifice group; (**E**) Lung injury and 72 h after sacrifice group; and (**F**) Lung injury with PDRN-treatment and 72 h after sacrifice group. **Lower**: The relative expressions of adenosine A_2A_ receptor (**left**), TNF-α (**middle**), and IL-6 (**right**) in each group. * represents *p* < 0.05 compared to the control and 24 h after sacrifice group. # represents *p* < 0.05 compared to the lung injury and 24 h after sacrifice group.

To verify the effect of PDRN on adenosine A_2A_ receptor, TNF-α, and IL-6 expressions, their relative expressions were ascertained (Figure 5). When the level of adenosine A_2A_ receptor (44 kDa) in the control and 24 h after sacrifice group was set at 1.00, the level of adenosine A_2A_ receptor was 0.29 ± 0.03 in the in the lung injury and 24 h after sacrifice group, 0.55 ± 0.02 in the lung injury with PDRN-treatment and 24 h after sacrifice group, 1.03 ± 0.10 in the control and 72 h after sacrifice group, 0.61 ± 0.07 in the lung injury and 72 h after sacrifice group, 0.95 ± 0.05 in the lung injury with PDRN-treatment and 72 h after sacrifice group.

When the level of TNF-α (26 kDa) in the control and 24 h after sacrifice group was set at 1.00, the level of TNF-α was 1.27 ± 0.09 in the in the lung injury and 24 h after sacrifice group, 1.01 ± 0.04 in the lung injury with PDRN-treatment and 24 h after sacrifice group, 0.70 ± 0.06 in the control and 72 h after sacrifice group, 1.54 ± 0.08 in the lung injury and 72 h after sacrifice group, 0.70 ± 0.04 in the lung injury with PDRN-treatment and 72 h after sacrifice group.

When the level of IL-6 (21 kDa) in the control and 24 h after sacrifice group was set at 1.00, the level of adenosine IL-6 was 1.24 ± 0.01 in the in the lung injury and 24 h after sacrifice group, 0.62 ± 0.15 in the lung injury with PDRN-treatment and 24 h after sacrifice group, 0.90 ± 0.08 in the control and 72 h after sacrifice group, 1.31 ± 0.06 in the lung injury and 72 h after sacrifice group, 0.66 ± 0.09 in the lung injury with PDRN-treatment and 72 h after sacrifice group. 

These results indicate that adenosine A_2A_ receptor expression was significantly decreased by the induction of lung injury (*p* < 0.05), whereas, TNF-α and IL-6 expressions were significantly increased in the lung tissues (*p* < 0.05). However, PDRN treatment significantly increased adenosine A_2A_ receptor expression (*p* < 0.05) and suppressed TNF-α and IL-6 expressions (*p* < 0.05).

## 3. Discussion

LPS is the most important pathogenic component that contributes to the development of ALI, and intratracheal instillation of LPS has been commonly used to induce an animal model of ALI [21,22]. Once LPS, an exogenous toxin, enters the bloodstream, it elicits systemic inflammation that mimics the initial clinical features of ALI [23,24]. In this model, LPS induces the early expression of inflammatory mediators, leukocyte accumulation, and apoptosis in the lung tissue, causing pulmonary edema and mortality [24,25,26]. 

Histological examination of ALI shows hemorrhage and edema [27]. Lung injury scores from histological analysis are commonly used to evaluate the severity of lung injury; pathological findings include alveolar capillary congestion, hemorrhage, infiltration, or aggregation of inflammatory cells in the airspace or interstitium, and thickening of the alveolar wall/hyaline membrane [24,28,29]. 

In the present study, intratracheal instillation of LPS produced a lung injury model in rats. Alveolar capillary congestion, hemorrhage, infiltration of inflammatory cells, and thickness of the alveolar walls were observed, and then lung injury score was assessed after intratracheal LPS instillation. 

Apoptosis is an important contributor to the aggravation of lung diseases, such as ALI and chronic obstructive pulmonary disease. Furthermore, the cellular environment of these acute and chronic lung diseases favors the delayed clearance of apoptotic cells [7,30]. Excessive apoptosis and/or deficient efferocytosis may affect lung disease outcomes [9,31,32]. Intratracheal instillation of LPS has been shown to increase inflammatory cytokines and apoptotic factors, such as caspases, Bax, and DNA fragmentation in the lung tissues, resulting in ALI symptoms [2,24]. Thus, excessive apoptosis plays a key role in the progression of ALI. 

In the present study, percentages of TUNEL-positive, cleaved caspase-3-, -8-, -9-positive cells, the ratio of Bax to Bcl-2, and expressions of inflammatory cytokines (TNF-α, IL-6) were increased following intratracheal LPS instillation, suggesting that LPS potentiated apoptosis. 

Activation of adenosine A_2A_ receptors in human monocytes and animal macrophages inhibits the secretion of cytokines [33]. Adenosine binds to the adenosine A_2A_ receptor, which attenuates apoptotic cell-induced nitric oxide formation and the consequent neutrophil chemoattractant induction through the activation of the adenylate cyclase pathway [34,35]. Jeon et al. [20] reported that PDRN, an adenosine A_2A_ receptor agonist, inhibited apoptosis in a gastric ulcer animal model. 

In the present study, the expression of the adenosine A_2A_ receptor was suppressed by intratracheal instillation, whereas PDRN treatment led to its overexpression in LPS-induced lung injury rats. These results indicate that PDRN potently activates the adenosine A_2A_ receptor in the lung injury.

Cyclic adenosine 3′,5′-monophosphate (cAMP) plays a key role in the modulation of cell death. When coupled with G-protein, adenosine A_2A_ receptor triggers or inhibits production of cAMP depending on the physiological conditions. Adenosine A_2A_ receptor activates production of cAMP [36]. In pulmonary epithelial cells, increment of cAMP by adenosine A_2A_ receptor inhibits apoptosis by release of anti-apoptotic proteins [34,37,38]. 

In the present study, percentages of TUNEL-positive, cleaved caspase-3-, -8-, -9-positive cells, the ratio of Bax to Bcl-2, and expressions of inflammatory cytokines were inhibited by PDRN treatment in LPS-induced lung injury rats. 

In conclusion, treatment with PDRN reduced lung injury score. This improving effect of PDRN on lung injury may be due to the enhanced effect of PDRN on adenosine A_2A_ receptor expression. These results demonstrate that PDRN treatment inhibits apoptosis and decreases lung injury score following lung injury. Based on this study, PDRN can be considered as a new remedy for the treatment of lung injury.

## 4. Materials and Methods

### 4.1. Animals and Grouping

Adult male Sprague-Dawley rats, weighing 250 ± 10 g (nine weeks old), were used for the experiments. All experimental procedures were carried out in accordance with the Guidelines for the Care and Use of Animals approved by the National Institutes of Health Council for management and use of laboratory animals. The study was approved by the Institutional Care and Use Committee of Kyung Hee University (KHUASP[SE]-16-026; 1 April 2016). The rats were housed under controlled temperature (23 ± 2 °C) and lighting (08:00 to 20:00, 12 h) conditions with food and water available ad libitum. The rats were randomly divided into six groups (*n* = 6 in each group) according to the sacrifice time and treatments: Control and 24 h after sacrifice group, lung injury and 24 h after sacrifice group, lung injury with PDRN-treatment and 24 h after sacrifice group, control and 72 h after sacrifice group, lung injury and 72 h after sacrifice group, and the lung injury with PDRN-treatment and 72 h after sacrifice group. 

### 4.2. Induction of Lung Injury and PDRN Treatment

The lung injury model was induced following the previously-described method [15,21]. After being anesthetized with Zoletil 50^®^ (10 mg/kg, i.p.; Vibac Laboratories, Carros, France), lung injury was induced in rats by intratracheal instillation of LPS (5 mg/kg, Sigma Chemical Co., St. Louis, MO, USA) in 0.2 mL saline; the control treatment consisted of intratracheal instillation of an equal volume of normal saline. Rats in the PDRN-treated groups intraperitoneal received a single injection of 0.3 mL distilled water including PDRN (8 mg/kg, Pharmaresearch Products Co., Ltd., Gyung-Gi Do, Korea), 1 h after lung injury. For the effective concentration of PDRN, preliminary experimental results and a previous study by Jeon et al. [20] were considered. Therefore, we used a dose of 8 mg/kg PDRN in this study.

### 4.3. Tissue Preparation

According to the previous described method [20,39], the rats were sacrificed at 24 h and 72 h after PDRN administration. The animals were anesthetized using Zoletil 50^®^ (10 mg/kg, i.p.; Vibac Laboratories), transcardially perfused with 50 mM phosphate-buffered saline (PBS), and the right lobe of the lung harvested. The lungs were fixed in 4% paraformaldehyde (PFA), dehydrated in graded ethanol, treated with xylene, infiltrated with paraffin, and embedded. A paraffin microtome (Thermo Co., Cheshire, UK) was used to make 5 μm thick coronal slices and the slices were placed on the coated slides. The slides were dried at 37 °C overnight on a hot plate. Six slice sections were collected from each lung sample.

### 4.4. Hematoxylin and Eosin Staining

H&E staining was conducted as the previous described method [20]. The slides were immersed in Mayer’s hematoxylin (DAKO, Glostrup, Denmark) for 30 seconds, rinsed with tap water until clear, dipped in eosin (Sigma Chemical Co., St. Louis, MO, USA) for 10 seconds, and again rinsed with water. The slides were air-dried at room temperature and then dipped twice in 95% ethanol, twice in 100% ethanol, twice in 50% ethanol, and 50% xylene solution, and twice in 100% xylene. Finally, coverslips were mounted using Permount^®^ (Fisher Scientific, Waltham, MA, USA). 

### 4.5. Analysis of Lung Injury Score

Lung injury scores were obtained with the previously-described method [24,29]. Images of H&E stained slides were taken with an Image-Pro^®^ plus computer-assisted image analysis system (Media Cyberbetics Inc., Silver Spring, MD, USA) attached to a light microscope (Olympus, Tokyo, Japan). Inspectors who did not know the identity of the slide evaluated the image. The sections were assessed for alveolar capillary congestion, hemorrhage, infiltration or aggregation of inflammatory cells in the airspace or interstitium, as well as the thickness of the alveolar wall/hyaline membrane formation. Each characteristic was scored from 0 to 3 (0 = absence; 1 = mild; 2 = moderate; 3 = prominent).

### 4.6. TUNEL Assay

TUNEL analysis was conducted with the previously-described method [20,39] using an In Situ Cell Death Detection Kit^®^ (Roche, Mannheim, Germany). The paraffin slides with embedded lung tissue were deparaffinized with xylene, rehydrated in graded ethanol, and rehydrated with running water for 5 min. The tissues were denatured for 10 min in boiling 10 mM citric acid (pH 6.0), and allowed to stand at room temperature for 10 min. The sections were post-fixed in ethanol-acetic acid (2:1), and then rinsed. The sections were then incubated with proteinase K (100 μg/mL), rinsed, incubated in 3% H_2_O_2_, permeabilized with 0.5% Triton X-100, rinsed again, and incubated in the TUNEL-reaction mixture. The sections were rinsed and visualized using Converter-POD with 0.05% 3,3′-diaminobenzidine (DAB). The slides were air-dried overnight at room temperature, and coverslips were mounted using Permount^®^ (Fisher Scientific, Waltham, MA, USA). 

### 4.7. Cleaved Caspase-3, -8, and -9 Immunohistochemistry 

Immunohistochemistry for cleaved caspase-3, -8, and -9 was performed with the previously-described method [13,20]. The paraffin slides with embedded lung tissue were deparaffinized in xylene, rehydrated in graded ethanol, and rehydrated in running water for 5 min. The tissues were denatured for 10 min in boiling 10 mM citric acid (pH 6.0), and allowed to stand at room temperature for 10 min. The sections were incubated overnight with rabbit anti-cleaved caspase-3, -8, and -9 antibodies (Cell Signaling Technology Inc., Danvers, MA, USA) at a dilution of 1:200. The sections were incubated for 1 h with biotinylated anti-rabbit secondary antibody (Vector Laboratories, Burlingame, CA, USA). The sections were subsequently incubated with avidin-biotin-peroxidase complex (Vector Laboratories, Burlingame, CA, USA) for 1 h at room temperature. Immunoreactivity was visualized by incubating the sections in a solution consisting of 0.05% 3,3-DAB and 0.01% H_2_O_2_ in 50 mM Tris-buffer (pH 7.6) for approximately 3 min. The slides were air-dried overnight at room temperature, and coverslips were mounted using Permount^®^ (Fisher Scientific, Waltham, MA, USA). 

### 4.8. Western Blot Analysis of Adenosine A_2A_ Receptor, Bax, Bcl-2, TNF-α, and IL-6

Western blot was conducted with the previously-described method [20,39]. Lung tissues were homogenized using lysis buffer containing 50 mM Tris-HCl (pH 8.0), 150 mM NaCl, 10% glycerol, 1% Triton X-100, 1.5 mM MgCl_2_·6H_2_O, 1 mM EGTA, 1 mM PMSF, 1 mM Na_2_VO_4_, and 100 mM NaF, then centrifuged at 10,000× *g* for 30 min. Protein content was measured using a Bio-Rad colorimetric protein assay kit (Bio-Rad, Hercules, CA, USA). Protein of 30 μg from each sample was separated on SDS-polyacrylamide gels and transferred onto a nitrocellulose membrane. Rabbit adenosine A_2A_ receptor antibody (1:1000; Abcam, Cambridge, UK), goat TNF-α antibody (1:1000; Santa Cruz Biotechnology, Dallas, TA, USA), goat IL-6 antibody (1:1000; Santa Cruz Biotechnology, Dallas, TA, USA), mouse β-actin antibody (1:1000; Santa Cruz Biotechnology, Dallas, TA, USA), mouse Bax antibody (1:1000; Santa Cruz Biotechnology, Dallas, TA, USA), and mouse Bcl-2 antibody (1:1000; Santa Cruz Biotechnology, Dallas, TA, USA) were used as the primary antibodies. Horseradish peroxidase-conjugated anti-mouse antibody (1:2000; Vector Laboratories, Burlingame, CA, USA) for β-actin, Bax, and Bcl-2, anti-goat antibody (1:2000; Vector Laboratories, Burlingame, CA, USA) for TNF-α, IL-6, and anti-rabbit antibody (1:3000; Vector Laboratories, Burlingame, CA, USA) for adenosine A_2A_ receptor were used as the secondary antibodies. Experiments were performed at room temperature except for membrane transfer. Membrane transfer was performed at 4 °C using a cold pack and pre-chilled buffer. Band detection was performed using an enhanced chemiluminescence (ECL) detection kit (Santa Cruz Biotechnology, Dallas, TA, USA). To compare the relative expressions of proteins, we used the Molecular Analyst^TM^ version 1.4.1 (Bio-Rad, Hercules, CA, USA) to calculate the detected bands.

### 4.9. Data Analysis

Data analysis was conducted with the previously-described method [20,39]. Histological observations were performed and percentages of TUNEL-positive and cleaved caspase-3-, -8-, -9-positive cells in lung tissue slices were calculated using an Image-Pro^®^ Plus computer-assisted image analysis system (Media Cyberbetics Inc., Silver Spring, MD, USA) attached to a light microscope (Olympus, Tokyo, Japan). For calculation of TUNEL-positive and cleaved caspase-3-, -8-, -9-positive cells, five visual fields were selected randomly from each sample and at least 100 cells per field were counted at 200× magnification. The percentages of TUNEL-positive and cleaved caspase-3-, -8-, -9-positive cells were calculated as follows: positive cells/total cells × 100 (%).

Statistical analysis was performed using one-way analysis of variance (ANOVA) and Duncan’s post-hoc test. The results were expressed as mean ± standard error of the mean (SEM). Significance was set at *p* < 0.05.

## Abbreviations

ALI	Acute lung injury
PDRN	Polydeoxyribonucleotide
LPS	Lipopolysaccharide
IL	Interleukins
Bax	Bcl-2-associated X protein
Bcl-2	B-cell lymphoma 2
Bid	BH3 interacting-domain death agonist
TNF-α	Tumor necrosis factor-alpha
TUNEL	Terminal deoxynucleotidyl transferase-mediated dUTP nick end labeling
H&E	Hematoxylin and eosin
cAMP	Cyclic adenosine 3′,5′-monophosphate
